# Downregulation of gastrokine-1 in gastric cancer tissues and restoration of its expression induced gastric cancer cells to apoptosis

**DOI:** 10.1186/1756-9966-31-49

**Published:** 2012-05-23

**Authors:** Wei Mao, Jie Chen, Tie-Li Peng, Xiao-Fei Yin, Lian-Zhou Chen, Min-Hu Chen

**Affiliations:** 1 Department of Gastroenterology, the First Affiliated Hospital of Sun Yat-Sen University, Guangzhou, China; 2 Department of Gastroenterology, the Third Affiliated Hospital of Sun Yat-Sen University, Guangzhou, China; 3 Digestive System Tissue Bank, the First Affiliated Hospital of Sun Yat-Sen University, Guangzhou, China

**Keywords:** Gastrokine-1, Gastric cancer, Immunohistochemistry, Cell viability, Apoptosis, 5-FU

## Abstract

**Background:**

Gastrokine-1 (GKN1), a secreted protein, is specifically expressed in gastric mucosa to protect and maintain the integrity of gastric epithelium. The present study investigated differential expression of GKN1 in normal, precancerous, and cancerous gastric tissues, and explored the biological functions of GKN1 protein in gastric cancer cells.

**Methods:**

RT-PCR, Western blot, and immunohistochemistry were performed to detect GKN1 expression in normal, precancerous, cancerous gastric tissues and seven gastric cancer cell lines. Gene transfection was used to restore GKN1 expression in gastric cancer AGS cells. Phenotypic changes (i.e., cell viability, apoptosis, cell cycle modulation, and sensitivity of gastric cancer cells to fluorouracil (5-FU)) were assayed in the transfected cells. DNA microarrays were used to analyze expression changes of apoptosis-related genes.

**Results:**

Significant downregulation or absence of GKN1 expression in seven gastric cancer cell lines were detected and progressive decrease of GKN1 expression from normal mucosa, precancerous tissue, to cancer tissues was observed. Moreover, restoration of GKN1 expression suppressed gastric cancer cell viability and induced the cells to undergo apoptosis. GKN1 expression also enhanced tumor cell sensitivity to 5-FU treatment. Moreover, it was found that GKN1 expression in AGS cells modulated expression of 19 apoptosis-related genes.

**Conclusions:**

Expression of GKN1 is progressively lost from normal mucosa, precancerous to cancerous gastric tissues, while restoration of GKN1 expression induces gastric cancer cells to undergo apoptosis, and enhances sensitivity of gastric cancer cells to 5-FU-induced apoptosis.

## Background

Gastric cancer is the fourth most common cancer, and one of the leading causes of cancer-related deaths in the world [1-3]. Although there have been a number of recent research advances in gastric cancer, such as improvements in early detection, advances in molecular research, newer surgery techniques, and more chemotherapy options, the overall survival rate for patients with gastric cancer has not been significantly improved. Therefore, research that focuses on new biological markers of early detection, new agents of possible genetic target, and further elucidating the molecular mechanisms will contribute to the therapeutic strategies.

Gastrokine-1 (GKN1), a novel protein cloned by a Japanese group in 2000 [4], is exclusively expressed in the gastric epithelium and easily biopsied. During gastric carcinogenesis, the GKN1 protein is downregulated in comparison to abundant expression in normal gastric mucosa [5]. Thus, this protein may be a potential biological marker for early detection of gastric cancer. Functionally, GKN1 promotes the maturation of gastric mucosa, and maintains the integrity of gastric mucosal epithelium through mitogenic and mutagenic abilities [6]. GKN1 may also protect the intestinal mucosal barrier by acting on specific tight junction proteins and stabilizing perijunctional actin [7]. Molecularly, the GKN1 protein contains a BRICHOS domain, which is associated with dementia, respiratory distress and cancer [8]. Therefore, the deficiency of GKN1 will result in the instability of gastric mucosa. The risk factors such as *H. pylori* can contribute to the down regulation of GKN1; meanwhile induce ulceration and cancer [9,10]. In addition, several studies observed that GKN1 expression was down regulation in gastric atrophy and intestinal metaplastic lesions and even absence in gastric cancer [5,11]. These studies demonstrate that GKN1 may play a key role in the gastric cancer progression.

In the present study, we examined GKN1 expression in tissue specimens of normal, premalignant, and malignant gastric mucosa. We then investigated the possible biological functions of GKN1 in gastric cancer cells by assessing the resulting phenotypic changes in GKN1 transfected cells. The primary aim of this study was to identify and characterize GKN1 as a potential tumor suppressor in gastric cancer.

## Methods

### Tissue specimens

Tissue specimens of atrophic gastritis, intestinal metaplasia, dysplasia, and gastric cancer were obtained from a total of 159 patients in our university hospitals. The premalignant lesions were from patients who underwent upper gastrointestinal endoscopy. Tissues of gastric tumors and their corresponding distant non-tumor tissues were collected from 39 gastric cancer patients who underwent surgery (Table 1). None of the gastric cancer patients received preoperative chemotherapy or radiotherapy. In addition, 20 healthy volunteers were also obtained for this study and these individuals visited our hospital for routine physical examinations and were confirmed to be negative for *H. pylori* infection by using ^13^C-urea breath test. All participants signed a written informed consent, and our Institutional Review Board approved the work. All tissue specimens were histologically re-confirmed by pathologists [12].

**Table 1:** Clinic and histological characteristics of the study population

**Histological type**	**Patient number**	**Gender**		**Age(yr) mean ± SD**
		**Male**	**Female**	
Healthy volunteers	20	10	10	44.6 ± 12.7
Atrophic gastritis	40	25	15	50.2 ± 10.8
Intestinal metaplasia	40	26	14	60.9 ± 13.5
Dysplasia	40	30	10	64.0 ± 11.4
Gastric cancer	39	23	16	53.0 ± 10.0

### Gastric cancer cell lines

Seven gastric cancer cell lines, MKN28, MKN45, AGS, N87, SNU 1, SNU 16 and KATO, were obtained from the Riken Cell Bank (Tsukuba, Japan) or the American Type Culture Collection (Manassas, VA, USA). Cells were cultured in RPMI 1640 medium containing 10% fetal bovine serum (Hyclone, Logan, USA), and maintained at 37°C in a humidified 5% CO_2_ atmosphere.

### RNA isolation and RT-PCR

Gastric tissue specimens were homogenized with an ultrasound homogenizer. Total RNA from tissues and tumor cells was isolated using the Qiagen RNeasy Mini Kit (Qiagen, Hilden, Germany) according to the manufacturer’s instructions. After quantification, RNA was reverse transcribed into cDNA using ReverTra Ace™ Kit (Toyobo Co., Osaka, Japan). The newly synthesized cDNA was then amplified by PCR with specific primers for the GKN1 gene (5^′^-TTTGCTGGACTTCTTGGA-3^′^ and 5^′^-TCGACTTTGTTTGGGTTG-3′) or β-actin, which was used as an internal control. PCR amplification was performed under the following conditions: an initial cycle at 94°C for 5 min, followed by 28 cycles at 94°C for 45 sec, 53°C for 30 sec, and 72°C for 1 min, with a final extension at 72°C for 7 min. PCR products were subsequently electrophoresed on a 1.5% agarose gel, and visualized under a UV transilluminator.

### Protein extraction and Western blot

Total cellular protein was extracted from tissue specimens and gastric cancer cells, using a lysis buffer containing a 1X protease inhibitor cocktail (Roche, Mannheim, Germany). Protein was quantified using the BCA Protein Assay Kit (Pierce Biotechnology, Rockford, USA). Equal amounts of protein were resolved by10% SDS-PAGE, and electroblotted onto polyvinylidene difluoride (PVDF) membranes. Membranes were then blocked in 5% non-fat milk overnight, and the next day, were incubated for 2 h with a 1:500 dilution of anti-GKN1 antibody (Abnova, Taipei, China) or a 1:1000 dilution of an antibody against beta-actin (Cell Signaling Technology, Danvers, USA,). After washed with phosphate buffered saline (PBS) three times and incubation for 1 h with the appropriate secondary antibody, enhanced chemiluminescence (Pierce Biotechnology, Rockford, USA) was used for protein visualization.

### Immunohistochemistry

Paraffin sections (4 μm thick) were prepared, deparaffinized in xylene, and then hydrated through graded series of ethanol concentrations. Antigen retrieval was performed by heating the sections for 10 min at 100°C in 0.01 M citrate buffer (pH 6.0), endogenous peroxidase activity was quenched with 3% H_2_O_2_ for 15 min, and nonspecific staining was reduced by incubating with a blocking serum for 10 min. The sections were then incubated with mouse anti-human GKN1 (1:300, Abnova) at room temperature for 2 h. Then, a 2-step detection method was used according to the manufacturer’s instructions (EnVision™ Detection Kit, Gene Tech Co., China) by incubation of the tissue with the ChemMate™ EnVision™/HRP for 30 min at room temperature. The reaction was visualized by the CheMate™ DAB plus Chromogen. Lastly, the sections were counterstained with hematoxylin solution. Negative controls were performed by staining with primary antibody.

The stained sections were evaluated and scored for staining intensity and% of staining under a light microscope, i.e., percentage of staining was documented as 0 (<5%), 1 (5%-25%), 2 (26%-50%), 3 (51%-75%), and 4 (>75%). Staining intensity was documented as 0 (no immunostaining), 1 (weak), 2 (moderate), and 3 (strong). The value of these two scores were added together to garner a final score for each case: a scale of 0 (score less than 2), 1+ (score range from 2 to 3), 2+ (score range from 4 to 5), and 3+ (score range from 6 to 7). Immunostaining was assessed by an experienced pathologist who was blinded to the clinical data of the patients.

### Construction of GKN1 expression vector for gene transfection

GKN1 cDNA was amplified from total RNA of the normal gastric mucosa using PCR. GKN1 CDS fragments with SalI and BamHI restriction sites were then inserted into the pBudCE4.1 vector (Invitrogen, Carlsbad, CA, USA) using a DNA ligation kit from TaKaRa (Dalian, China). After transformation into DH5α *E. Coli* competent cells, the plasmid was amplified and the DNA sequence was then confirmed. To generate gastric cancer cells expressing GKN1, gastric cancer AGS cells were grown to 50–75% confluency in a six-well plate, washed twice with RPMI lacking supplements (RPMS/LS), and subjected to the Lipofectamine-mediated transfection according to the manufacturer’s protocol (Invitrogen). The GKN1 transfected gastric cancer cells were then selected in medium containing Zeocin (Invitrogen). After the transfected cells formed individual cell colonies, stable cells were obtained and then confirmed for GKN1 expression by using RT-PCR and Western blot analyses.

### Cell viability (MTT) assay

To detect changes in tumor cell viability after GKN1 transfection, a total of 1 × 10^4^ trypsin-dispersed cells in 0.1 mL culture medium was seeded into each well of a 96-well plate, and cultured for 24 h or 48 h. Next, 20 μL of MTT (5 g/L from Sigma-Aldrich, St. Louis, USA) was added to each well and incubated for additional 4 h at 37°C. Culture medium was then replaced with 200 μL of dimethyl sulfoxide (DMSO) and the absorbance rate was determined using an ELISA reader at 490 nm. Cell growth inhibition rate was calculated as (the value of experimental group OD /the value of control group OD) × 100%.

### Annexin V apoptosis assay

To detect tumor cell apoptosis, the GKN1 transfected tumor cells were seeded into 60-mm diameter culture plates, and cultured for 24 h and 48 h. The apoptotic rates were analyzed by flow cytometry using an annexin V-FITC/PI kit. Staining was performed according to the manufacturer’s instructions, and flow cytometry was conducted with a flow cytometer (Beckman-Coulter, Brea, USA). Cells with annexin V (−) and PI (−) were deemed viable cells. Cells with annexin V (+) and PI (−) were deemed early apoptotic cells. Cells with both annexin V (+) and PI (+) were deemed late apoptotic cells.

### TUNEL assay

To identify apoptosis in the transfected cells, we utilized the dead-end colorimetric TUNEL system kit (Promega, Madison, USA) to measure DNA fragmentation and caspase-3 activation in the GKN1 transfected cells, according to the manufacturer’s instructions. Briefly, cells were fixed in 4% paraformaldehyde solution for 25 min at room temperature, rinsed in PBS, and permeabilized by incubating the slides in 0.2% Triton X-100 solution. Cells were then incubated with a terminal deoxynucleotidyl transferase (TdT) reaction mixture containing biotinylated nucleotides and TdT at 37°C for 60 min, and rinsed with 1 × SSC (sodium chloride-sodium citrate buffer) and PBS. Next, streptavidin HRP was added to the cells, and the cell slides were stained with 3,3′-diaminobenzidine color solution. Finally, cells were examined under a light microscope and the number of positive cells was counted and summarized from a total of 10 high power fields.

### Cell cycle analysis

To analyze cell cycle distribution, transfected cells were grown and treated with 25 M olomoucine (Santa Cruz Biotechnologies, Santa Cruz, USA) for 1 h, and then incubated with regular culture medium for an additional 1 h [13]. Cells were then collected and subjected to cell cycle analysis by flow cytometry as described in the previous section.

### Sensitivity to 5-FU treatment

To detect the role of GKN1 in mediating sensitivity of gastric cancer cells to 5-FU treatment, we grew and treated GKN1 transfected tumor cells with 5-FU (Sigma) or DMSO for 24 h and 48 h. Concentrations of 5-FU ranged from 0.25 to 1.0 mmol/L. The apoptosis rate from these cells was detected by flow cytometry as previously described.

### cDNA microarray analysis

To perform cDNA microarray analysis, total cellular RNA from GKN1-transfected and vector-control tumor cells were isolated with the Trizol® Reagent (Invitrogen). RNA was then reversely transcribed into cDNA using the TrueLabeling-AMP Linear RNA amplification kit (Superarray, Frederick, MD, USA), and then converted into biotin-labeled cRNA using biotin-16-UTP and an in vitro transcription kit (Roche, Basel, Switzerland). The newly synthesized cRNA probes were then purified with the ArrayGrade cRNA cleanup kit (Superarray) and then added to the pretreated Oligo GEArrays Human Apoptosis Microarray (OHS-012 from Superarray) that contains 112 apoptosis-related genes. The microarray was then hybridized overnight at 42^o^C. The next day, the hybridized arrays were washed and detected by chemiluminescence according to the manufacturer’s instructions (Pierce). The data were analyzed using GEArray Expression Analysis software (Superarray). If spot intensity increased by more than two fold, this gene was deemed upregulated. In contrast, if spot intensity decreased by more than two fold, the gene was deemed downregulated.

### Statistical analysis

All quantitative data were expressed as mean ± SD and analyzed using Student *t*-tests. The differential expression of GKN1 among different groups was determined by Kruskal-Wallis test. All statistical analyses were performed using the SPSS statistical software package (version 11.0, SPSS Inc. Chicago, USA). A *P* value of < 0.05 was consi-dered statistically significant.

## Results

### Expression of GKN1 in cancer cell lines and gastric tissue specimens

**Figure 1:**
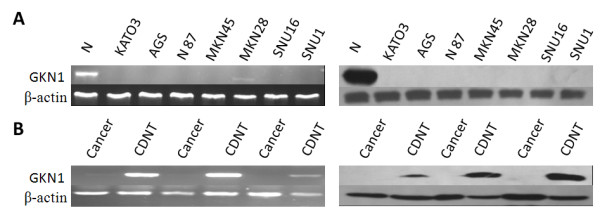
**Down regulation of GKN1 in gastric cancer cell lines and gastric tissue specimens.** GKN1 RNA and protein were extracted from tumor cell lines and gastric tissue samples and then subjected to RT-PCR and Western blotting analysis. **A**: GKN1 expression in gastric cancer cell lines. GKN1 mRNA and protein were absent in the cell lines except for mRNA was weakly expressed in MKN28 cells. Normal gastric mucosa (N) was also detected as control group. **B**: GKN1 expression in gastric tissue specimens. Expression of GKN1 mRNA and protein were significant down-regulated or even absent in gastric cancer tissues but abundant in the corresponding distant non-cancerous tissues (CDNT).

We first performed RT-PCR and immunoblot analysis to detect expression of GKN1 mRNA and protein levels in cancer cell lines and tissue specimens. We found that GKN1 mRNA was weakly expressed in gastric cancer MKN 28 cells, and was absence in AGS, N87, MKN45, SNU16, SNU1, and KATO cells (Figure 1A). The GKN1 protein was also not detectable in any of the seven cell lines (Figure 1A). In contrast, GKN1 mRNA and protein were abundance in normal gastric epithelial cells that were obtained from healthy volunteers (Figure 1B). In 39 gastric cancer tissues, GKN1 mRNA was only weakly expressed in 3 tissues, and absence in the remaining 36 tissues. GKN1 protein was weakly expressed in 2 gastric cancer tissues, and absence in the remaining 37 tissues. However, GKN1 mRNA and protein were abundantly expressed in all of the 39 corresponding distant non-cancerous tissues (Figure 1B).

**Figure 2:**
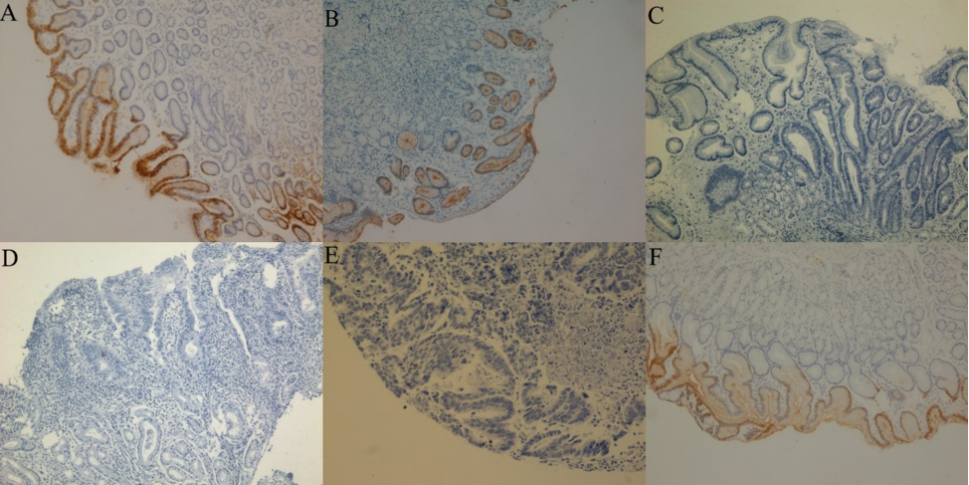
**Immunohistochemical detection of GKN1 protein in gastric tissue specimens.** Paraffin sections were immunostained with anti-GKN1 antibody and reviewed for GKN1 levels. GKN1 progressively decreased from normal gastric mucosa, atrophic gastritis, intestinal metaplasia, and dysplasia to gastric cancer. **A**: normal gastric mucosa; **B**: atrophic gastritis; **C**: intestinal metaplasia; **D**: dysplasia; **E**, gastric cancer; **F**, the corresponding distant non-cancerous tissue.

Next, we immunohistochemically stained GKN1 in the tissue sections of normal gastric mucosae (from healthy volunteers), atrophic gastritis, intestinal metaplasia, dysplasia, and gastric cancer and their corresponding distant non-cancerous mucosae. We found that the GKN1 protein was abundantly expressed in the upper glandular layer of the top one third superficial epithelium, while expression of GKN1 protein was progressively down regulated from normal gastric mucosa, atrophic gastritis, intestinal metaplasia and dysplasia, to gastric cancer (Table 2) (Figure 2). This reduction in expression was statistically significant (p < 0.05).

**Table 2:** GKN1 expression detected by immunohistochemistry in gastric tissues

**Histological type**	**Number of patient**	**-**	**+**	**++**	**+++**	**P value**^1^
Normal gastric mucosa	20	0	0	0	20	< 0.05
Atrophic gastritis	40	0	15	20	5	
Intestinal metaplasia	40	21	19	0	0	
Dysplastic lesion	40	26	14	0	0	
Gastric cancer	39	34	5	0	0	

^1^Comparison between healthy volunteers, atrophic gastritis, intestinal metaplasia, dysplasia and gastric cancer tissues, P < 0.05.

### Transfection of GKN1 reduced gastric cell proliferation

**Figure 3:**
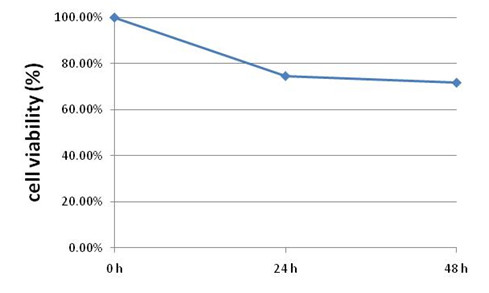
**Suppression of cancer cell viability by GKN1.** The GKN1 or vector transfected gastric cancer cells were grown and subjected to MTT assay. The data showed that viability of AGS cells with GKN1 transfection was significantly decreased compared to the cells with vector transfection in 24 h (74.6%) and 48 h (71.7%).

Next, we determined whether restoration of GKN1 expression would suppress gastric cancer AGS cells viability. To this end, we generated AGS cells that stably expressed GKN1 expression was confirmed by RT-PCR and Weston blotting. Cell viability (MTT) assays showed that AGS cells stably expressing GKN1 grew at a much slower rate compared to the vector-transfected control cells in both 24 hour and 48 hour cultures (Figure 3). This data clearly indicate that restoration of GKN1 expression inhibits AGS cell proliferation.

### Effect of GKN1 on AGS cell apoptosis and cell cycle re-distribution

**Figure 4:**
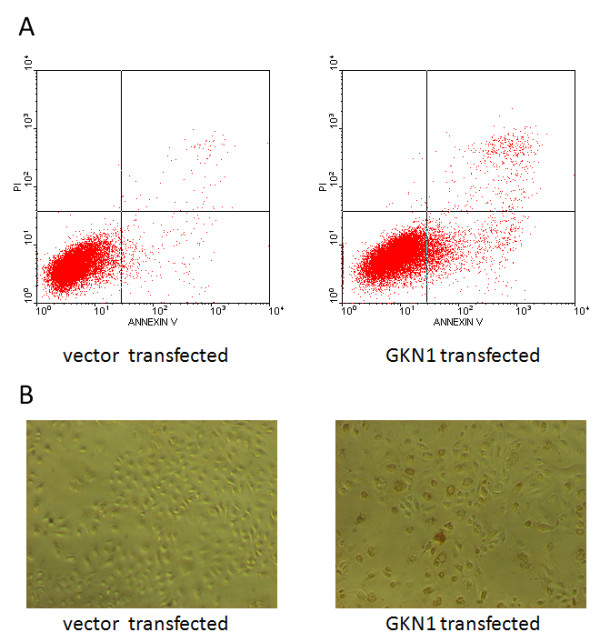
**Apoptosis induction of gastric cancer cell by GKN1.A**: Flow cytometric assay. The GKN1 or vector transfected gastric cancer AGS cells were grown and subjected to flow cytometry assay for detection of apoptosis; **B**: TUNEL assay. The GKN1 or vector transfected gastric cancer cells were grown on glass slides and then subjected to TUNEL assay.

We examined whether inhibition of cell proliferation by GKN1 was due to the induction of apoptosis. To this end, we examined the levels of apoptotic cells using flow cytometry, and found that compared to the vector transfected cells, GKN1 transfected AGS cells were apoptotic (Figure 4A). The TUNEL assay demonstrated that endogenous GKN1 significantly induced apoptosis in AGS cells, and examination of morphology demonstrated that the nuclei of GKN1 transfected tumor cells exhibited condensation and fragmentation (Figure 4B).

**Figure 5:**
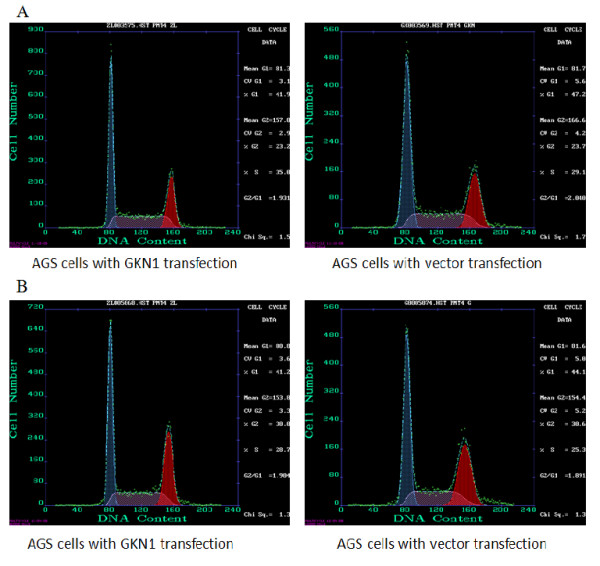
**Effect of GKN1 on cell cycle re-distribution.** The GKN1 or vector transfected AGS cells were arrested in the cell cycle with 1 h olomoucine treatment and continued to incubate for another 1 h without olomoucine. **A**: after 1 h olomoucine treatment; **B**: an additional hour incubation without olomoucine.

Next, we examined cell cycle changes in these tumor cells, because suppression of cell viability is closely related to regulation of the cell cycle. Olomoucine, a purine derivative, is a cyclin-dependent kinase (CDK) inhibitor, thus we used it to enrich parental AGS cells in the G1 phase. Specifically, cells were arrested in the cell cycle with 1 h olomoucine treatment and continued to incubate for another 1 h without olomoucine. The cell cycle distribution of GKN1 transfected cells changed from 41.9% of G1 and 35.0% of S phase to 41.2% of G1 and 28.7% of S phase of the cell cycles. Similarly, the cell cycle distribution of vector-transfected cells changed from 47.2% G1 and 29.1% of S phase to 44.1% G1 and 25.3% of S phase of the cell cycles (Figure 5). These data demonstrate that GKN1 is unable to arrest AGS cells in the G1-S transition phase of cells.

### GKN1 enhanced tumor cell sensitivity to 5-FU mediated apoptosis

**Figure 6:**
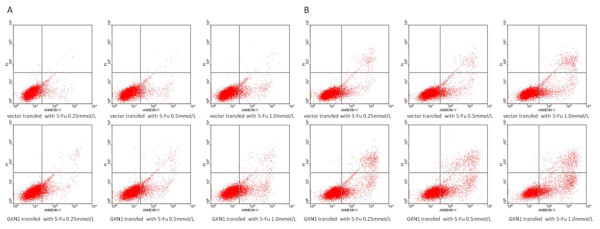
**GKN1 enhanced tumor cell sensitivity to 5-FU-mediated apoptosis.** The GKN1 or vector transfected gastric cancer cells were grown and treated with different doses of 5-Fu in 24 and 48 h. After that, these cells were subjected to flow cytometry assay for apoptosis. **A**: 5-Fu treatment for 24 h; **B**: 5-Fu treatment for 48 h.

Clinically, 5-FU is routinely used in the treatment of gastric cancer. In this study, we assessed whether presence of GKN1 could enhance sensitivity of gastric cancer cells to 5-FU treatment. Flow cytometry was used to detect apoptosis rate after 24 hours and 48 hours (Table 3) with different concentrations of 5-FU in the GKN1 transfected cells. The results showed that apoptosis was significantly induced in GKN1 transfected cells, in a time and dose-dependent manner, compared to the vector transfected cells (Table 3; Figure 6).

**Table 3:** 5-FU induction of apoptosis in gastric cancer AGS cells

**Group**	**Time (h)**	**5-FU-induced apoptosis (%)**		
		**0.25 mmol/L**	**0.5 mmol/L**	**1.0 mmol/L**
Vector transfected	24	5.53 ± 0.06	7.70 ± 0.10	9.57 ± 0.21
GKN1 transfected	24	13.03 ± 0.40	14.93 ± 0.15	19.73 ± 0.23
Vector transfected	48	8.23 ± 0.21	12.33 ± 0.21	14.33 ± 0.06
GKN1 transfected	48	18.13 ± 0.72	23.30 ± 0.79	34.83 ± 0.67

### GKN1 modulation of apoptosis-related gene expression

So far, we had demonstrated that GKN1 expression was able to induce apoptosis in gastric cancer cells. We therefore profiled the expression change of apoptosis-related genes in GKN1 transfected and vector transfected AGS cells by cDNA microarray. The Oligo GEArray-Human Apoptosis Microarray (OHS-012 from Superarray) contains 112 apoptosis-related genes. After hybridization of RNA probes from GKN1 or vector transfected AGS cells to the array, we could detect differential expression of these genes between GKN1 transfected and control cells. Specifically, a total of 16 genes were downregulated, and 3 genes were upregulated after restoration of GKN1 expression in AGS cells compared to the control cells (Table 4).

**Table 4:** Changed expression of apoptosis-related genes in GKN1-transfected AGS cells

**Gene symbol**	**GenBank number**	**Fold change**
ABL1	NM_005157	0.481
APAF1	NM_001160	0.489
BAX	NM_004324	0.347
BCL10	NM_003921	0.465
BCL2L1	NM_138578	0.257
BCLAF1	NM_014739	0.497
BOK	NM_032515	0.429
CARD11	NM_032415	0.181
CIDEB	NM_014430	0.366
NOL3	NM_003946	0.219
TNFRSF10C	NM_003841	0.365
TNFRSF10D	NM_003840	0.259
TNFRSF1A	NM_001065	0.358
TNFRSF6B	NM_003823	0.465
TP53BP2	NM_005426	0.381
TRAF3	NM_003300	0.478
BCL2A1	NM_004049	2.036
BCL2L11	NM_006538	2.267
CARD8	NM_014959	2.589

## Discussion

In the current study, we investigated expression of GKN1 mRNA and protein in tissue specimens from normal gastric mucosa, atrophic gastritis, intestinal metaplasia, dysplastic lesions, and gastric cancer. We found that GKN1 expression was progressively downregulated and lost from precancerous to cancerous tissues, indicating that the loss of GKN1 expression may contribute to gastric carcinogenesis. Previous studies showed decreased GKN1 expression in gastric cancer [5,14]. Our current study, for the first time, demonstrated the progressive loss of GKN1 mRNA and protein from normal to precancerous and cancer tissue specimens, indicating the role of GKN1 in gastric cancer homeostasis and alteration of GKN1 expression in gastric cancer.

To further investigate the possible biological functions of GKN1 in gastric cancer, we successfully cloned and transfected GKN1 into gastric cancer AGS cells that do not express GKN1 protein. We found that restoration of GKN1 expression suppressed tumor cell viability and induced them to undergo apoptosis and enhanced effects of 5-FU on gastric cancer cells. These data indicate the role of GKN1 in gastric cancer and could be further developed as a novel target for control of gastric cancer.

The following data of flow cytometry and TUNEL assay showed that GKN1 may induce apoptosis in cancer cells. These data were consistent with the previous studies [15,16]. The regulation of cell cycle redistribution closely correlated with suppression of cancer cells. After GNK1 transfected, AGS cells were treated with olomoucine, a CDK inhibitor, to enrich cells at G1 phase of the cell cycle. But GKN1 was unable to hold cells in the G1-S transition phase, suggesting that GKN1 may not affect the cell cycle. Nevertheless, other studies found that overexpression of GKN1 resulted in cell cycle arrest at G1 phase [17] or G2/M phase of the cell cycles [18]. The reason for this discrepancy is unclear, but may be because that the exogenous GKN1 protein was not equal to the endogenous protein in regulation of cell phenotypes or functions. Our current study using the gene transfection technique demonstrated that induction of GKN1 expression induced apoptosis of gastric cancer AGS cells. However, further studies are needed to explore this discrepancy.

Both the previous studies [5,9] and our current immunohistochemical data showed that the GKN1 protein was expressed in the top layers of gastric mucosa and glands, but was absent in the deeper layer of the mucosa and glands. This localization may contribute to the mitogenic and restitutional functions of GKN1 protein in maintenance of gastric mucosa homeostasis [19]. It is because the top layers of the epithelium are committed to apoptosis process in physiological condition. However, other studies suggested that GKN1 may be secreted from epithelial cells, and have functions in both paracrine and autocrine systems [6] in control of normal cell growth, differentiation, and apoptosis.

In addition, this study demonstrated that GKN1 was able to increase the sensitivity of gastric cancer cells to 5-FU treatment. This finding suggested that GKN1 may be useful as an adjuvant target in combination with other chemotherapeutical agents in the treatment of gastric cancer. 5-FU has been a widely used as a chemotherapeutic agent in treating patients with gastric cancer. It is a pyrimidine analogue and can incorporate into DNA or RNA for the induction of cell cycle arrest and apoptosis through inhibition of DNA duplication in tumor cells. In this regard, GKN1 could induce cell apoptosis, thus GKN1 could enhance 5-FU antitumor activity in gastric cancer cells. This result may partially explain the reason that patients who have lost GKN1 expression have shorter overall survival [20]. However, it remains to be determined how GKN1 is able to induce apoptosis in gastric cancer cells.

Our preliminary data revealed that GKN1 expression was able to modulate expression of several apoptosis-related genes using a cDNA microarray analysis. Of the 112 genes covered by the Oligo GEArrays Human Apoptosis Microarray, the expression of 19 genes may directly affect by GKN1. However, some of these screening genes (such as BAX and BCL2A1) may be indirectly or even not affected or regulated by GKN1 protein [14,21]. Considering limitations of the microarray analysis, these screening genes need to be verified by qRT-PCR or western blot analyses in the further study.

## Conclusions

In summary, expression of GKN1 mRNA and protein was progressively downregulated from the normal mucosa, precancerous to cancerous gastric tissues. Restoration of GKN1 expression induced gastric cancer cells to undergo apoptosis, and enhanced sensitivity to 5-FU-induced apoptosis. These data indicate that GKN1 plays a role in regulation of gastric epithelial homeostasis and that lost GKN1 expression could contribute to gastric cancer development.

## Abbreviations

GKN1: Gastrokine-1; 5-FU: 5-fluorouracil.

## Competing interests

The authors declare that they have no competing interests.

## Authors^’^ contributions

Mao W and Chen J performed the experiments and wrote the paper; Peng TL and Yin XF organized the figures and collected tissue specimens and patients’ data; Chen MH designed this study and supervised the writing and discussion. All authors have read and approved the final version of this manuscript.
